# Bridging the Gaps: Bole and Terra Sigillata as Artefacts, as *Simples* and as Antibacterial Clays

**DOI:** 10.3390/min10040348

**Published:** 2020-04-14

**Authors:** Danae Venieri, Iosifina Gounaki, George E. Christidis, Charles W. Knapp, Petros Bouras-Vallianatos, Effie Photos-Jones

**Affiliations:** 1 School of Environmental Engineering, Technical University of Crete, 73100 Chania, Greece; 2 School of Mineral Resources Engineering, Technical University of Crete, 73100 Chania, Greece; 3 Civil and Environmental Engineering, University of Strathclyde, Glasgow G1 1XQ, UK; 4 School of History, Classics and Archaeology, University of Edinburgh, Edinburgh EH8 9JX, UK; 5 Analytical Services for Art and Archaeology (Ltd.), Glasgow G12 8JD, UK; 6 Archaeology, School of Humanities, University of Glasgow, Glasgow G12 8QQ, UK

**Keywords:** Armenian bole, Terra Sigillata, antibacterial clays, Lemnian Earth, Terra Tripolitania, medicinal earths

## Abstract

Medicinal earths are an important and yet, so far, little scientifically explored archaeological resource. They are almost always identified by their source locality. Our work over the last few years has focused on their chemical and mineralogical characterization and their testing as anti-bacterials. This paper presents the results of the mineralogical analysis and antibacterial testing of six medicinal earths, *bole* or *Terra Sigillata* (stamped earth) of unknown date and provenance in the Pharmacy Museum of the University of Basel. Only one of them, a red (Armenian?) ‘bole’, was found to be antibacterial against both Gram-positive and Gram-negative bacteria. A yellow powder of Terra Tripolitania was mildly antibacterial and against one pathogen only. We argue that medicinal earths are in a pivotal place to bridge the gap between currently dispersed pieces of information. This information relates to: (a) their nature, attributes, and applications as described in the texts of different periods, (b) the source of their clays and how best to locate them in the field today, and (c) the methods employed for their beneficiation, if known. We propose that work should be focused primarily onto those medicinal earths whose clay sources can be re-discovered, sampled and assessed. From then on, a parallel investigation should be initiated involving both earths and their natural clays (mineralogy at bulk and nano-sized levels, bio-geochemistry, microbiological testing). We argue that the combined study can shed light into the parameters driving antibacterial action in clays and assist in the elucidation of the mechanisms involved.

## 1 Introduction

Medicinal earths, (Latin: *terras*, Greek: *gaies*), *boles* or *Terra Sigillata* (stamped earth) were pharmacological agents for diverse ailments, frequently mentioned in the texts of ancient authors of classical and late antiquity (for example, Dioscorides, Pliny and Galen). The use of at least some of them continued well into the early 20th century. They were ingested or applied externally, on their own (*as simples*) or in compound drugs in association with botanicals and/or animal products. They were characterized as astringents and drying agents and described generically, as antidotes to ‘poison’.

Many institutions, public and private, hold cabinets or collections of these medicinal earths, some stamped with the name of their place of origin, and referred to as Terra Sigillata, or sealed earth. They provide material proof of the earths mentioned in the texts, but also the clays of the localities from where they are purported to originate [1]. The difference between bole and Terra Sigillata was already highlighted in the 18th century by Hill [2]. He defines the former as ‘less compact than clays but more so than marls’ (consisting of clay and lime). In the Swiss pharmacopoeia of 1893 (3rd edition, German version), Zurich unstamped clays were referred to as bolus. Two types were recognised; one was white and called Bolus alba, the other red, usually of Armenian origin and called Bolus Armenaeus rubra.

Perhaps the first and most famous amongst the stamped earths was Terra Lemnia (Lemnian Earth) from the island of Lemnos in the N.E. Aegean with a recorded longevity of use from the Classical/Hellenistic period until the early 20th century [3–6]. When the island came under Ottoman rule in the 16th century, its extraction and distribution was strictly administered by the authorities in Istanbul. Its scarcity in the markets of Europe led to a drive to find other medicinal earths which could fill the demand for antidotes to ‘poison’ and preventives ‘against the plague’. It is in that context that other earths began to emerge in Central Europe, like Terra Silesia (or Silesiaca), in modern day Poland [7]. Other sources included Terra Melitea (Malta), Livonia (Baltic), Terra Sigillata from many parts of France, English boles, Earth of Ireland, Terra Florentina (Italy), Terra Hispanica and Portugalica; in the Middle East there was Terra Turcica, Armenica, and Hierosolymitanae, all clearly denoting place of origin, albeit in very broad geographical terms [8]. However, confidence as to the origin and, by association, efficacy of the stamped pellets circulating in the markets was being undermined already from the early 18th century; ‘everyone makes ’em to his Fancy’ wrote Pomet [9] (as quoted in [8] (p. 114)). Lemnian Earth was the first to be tested chemically [10]. Nearly a hundred years later, De Launay [11] (p. 318) analyzed samples retrieved from the pit of its extraction but found it to be no more than just ‘clay’. In a pensive mode he wrote: ‘the failure of my attempts is proof of what happens when one is trying to apply chemistry to myths’ (quoted in [6] (p. 458)). Thomson [12] carried out analysis on samples of 16th century troches or stamped pellets of Lemnian Earth with the same results, concluding that it was a clay with no pharmacological value (for a detailed presentation of early Lemnian Earth chemical analyses see MacGregor [8]).

**Figure 1:**
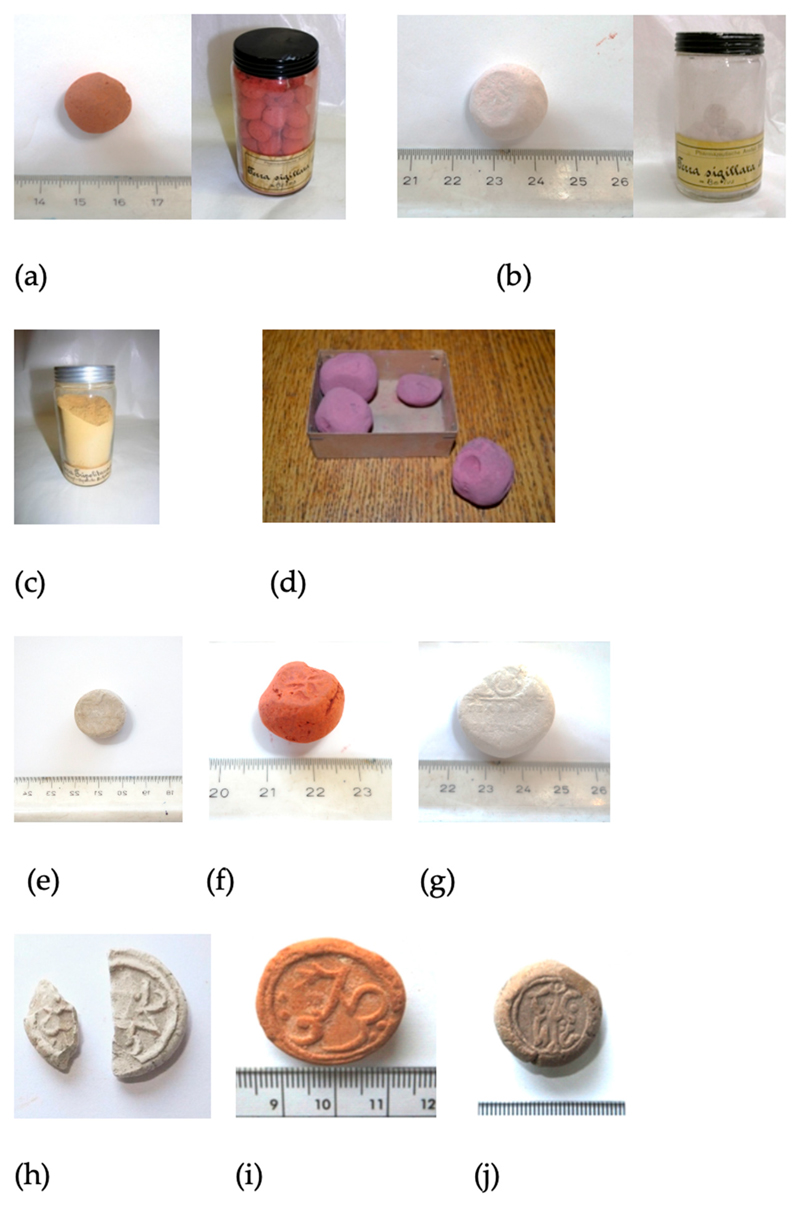
(a) Z-01405. Left: sample received, with no seal. Right: Terra Sigillata rubra, also with no stamped seals; with a label marked: Pharmaceutical Institution, Basel; (b) Z-01406). Left: sample as received. Right: Terra Sigillata alba = bolus; with a label marked: Pharmaceutical Institution, Basel; (c) Z-O1277. Powdered sample; with label marked: Alte Engel-Apotheke St.Gallen (old Angel Pharmacy, St Gallen); (d) Z-1628. Four small samples of purple Terra Sigillata—one sample was analyzed but with inconclusive results so they are not reported here: (e) Z-01627. Terra Sigillata without stamp; (f) Z-01629-1 and (g) Z-01629-2; Terra Sigillata, with faded stamp; label on container bearing the logo of Wespi Pharmacy, Brugg; (h) sample 700.4 (white), (i) 700.17 (red), and (j) 700.18 (yellow-grey), all Lemnian Earths from the collection of the University of Basel Museum of Pharmacy (after Photos-Jones et al. [15]).

Our work over the past twenty years and on the purported place of its extraction, in Kotsinas in the north-east of Lemnos, has resulted in sampling local deposits of sedimentary clays and the waters of local springs [13,14]; the latter seem to have played a role in the ‘preparation’ of the earth, at least as hinted by the visitors to the island in the Ottoman period [14]. In the last three years we have also undertaken analysis and preliminary (qualitative) antibacterial testing of pellets of Lemnian Earth (*sphragides*) from the collection of the Pharmacy Museum of the University of Basel [15] (see also Figure 1h,j). More recently, quantitative assessment of the same samples showed that two of the three artefacts were antibacterial against both Gram-negative and Gram-positive pathogens, but the third was mildly so and against only one of the two bacteria tested [16].

For purposes of clarification, in this paper, we refer to the museum artefacts as ‘earths’ or ‘stamped earth’ or ‘bolus’ to denote the final product or ‘medicine’ and to differentiate them from the raw material, the ‘clay’. By ‘clay’ we refer to the mixture of clay and non-clay minerals that made up the ‘earths’, and which was almost always subjected to some kind of beneficiation. Earths are ‘medicinal’ and were perceived as such for a number of reasons, for example as adsorbants of toxins [7].

In this paper we present the results of the mineralogical and microbiological analysis of six earths in the collection of the of the Pharmacy Museum of the University of Basel, of unknown date and provenance (Figure 1a–g). The three samples of 16th–18th century Lemnian Earth are included here both for purposes of illustration and comparison of analytical results (see Discussion) (Figure 1h,j). Regarding the six samples, the Museum’s catalogue refers to these earths as Terra Sigillata (stamped earth) but, in fact, few were stamped and, when they were stamped, the stamp was nearly illegible. Therefore, their provenance and date cannot be known for certain. Apart from Terra Sigillata, two additional descriptors are given and for two of the six samples only: sample 01277 (Figure 1c) was referred to as Terra Tripolitania, presumably from a locality in N.W. Libya’s region of the same name, and sample 01406 (Figure 1b) was labelled Terra Sigillata alba = bolus.

Among the Dutch, German, and Swiss texts of the 17th to the 19th centuries, references to red and white medicinal clays, usually termed bolus Armenicus or rubrus and bolus albus respectively, give a reasonably consistent picture of their medicinal properties. For example, Schröder’s pharmacopoeia [17] mentions rubrica as an astringent, drying agent with strengthening properties, used for various wounds, bleeding, anything requiring absorption and, externally, for poultices. In a lexicon of 1721, Lemery [18] refers to the red bolus being used internally for diarrhea (and similar intestinal ailments) and bloody coughs; good for reducing acidity, and externally it is a suitable styptic (Ianto Jocks, pers. comm.). The white clay dries and cleans, and externally it is applied to wounds and ulcers. On the other hand, Albrecht von Haller’s *Pharmacopoeia Helvetica* of 1771 [19] (p. 43, 2nd vol.) mentions its use for fevers, epidemics, or vomiting, but does not mention external use.

The first notable description of Armenian bolus as a simple (one-ingredient drug) appears in Galen [20] (pp. 189.7-14, 192.1-3). It was described as yellowish (chroan ōchra) in color and was designated as a stone (lithos) by the person who gave it to Galen, although Galen himself believed that it would be better categorized as a type of earth (gē), since it formed a paste when mixed with water. It was soft and easily ground without any coarse components. Dioscorides [21] (pp. 63.16–19) described the so-called armenion as blue in colour (chroian kyaneon) and similar to chrysokolla in action. Armenian bole was used for various kinds of stomach ailments, for mouth ulcers, and was administered to those suffering from breathing difficulties [20] (p. 190).

It was also recommended for the treatment of infectious diseases causing epidemics (i.e., bubonic plague, typhoid fever, smallpox). Galen mentions that it was used in the Antonine Plague in Rome (AD 165–180), but the exact nature of that epidemic is not clear [20] (p. 191). The 6th-century physician Alexander of Tralles states that Armenian bole was effective for ‘quartan fever’, most probably one of four types of malaria. It was either used in the ‘as-washed’ state or as ‘unwashed’. It was reported as effective at ‘evacuating black bile better than any other agent’. If washed, it purged the lower bowels, while unwashed it acted as a vomiting agent ([22] (pp. 429.16-21); [23] (p. 227)). Furthermore, Alexander of Tralles mentions that the original Armenian bole was more effective than the Samian and Lemnian Earth [24] (pp. 207.13-14). The reference to ‘quartan fever’ in the latter author’s text is significant for the reasons outlined in the Discussion.

In this paper we present the results of the mineralogical (X-ray diffraction) analysis and the microbiological testing of the six earths against one Gram-positive and one Gram-negative pathogen to assess their relative antibacterial strengths and to compare them with similar results from previous work and with two samples of Lemnian Earth, in particular. Furthermore, we undertook DNA sequencing work on two samples 01405 and 01406, one bioactive and the other non-bioactive, respectively. The investigation aimed to determine the type of microorganisms present and, if present, whether they contributed to the bioactivity of the one of the two samples.

Bioactivity would be imparted via the production and excretion of secondary metabolites. Secondary metabolites are chemical compounds, not essential for growth, but exuded by the microorganisms themselves when under stress and for the purpose of aiding the organism’s survival. They can achieve the latter by reducing competition from other microorganisms or by detoxifying their immediate environment. They are often water-soluble or volatile and affect other organisms at a distance [25]. Secondary metabolites can be anti-oxidants, sequestrants (they form chelate compounds with metal ions and thus remove the latter from solution), antimicrobials or redox regulators reacting to chemical stressors, (for example, free radicals or reactive oxygen species). Chemical detection of secondary metabolites is quite complex analytically. Before embarking on such work, it is therefore essential to ascertain, via their residual DNA signatures, that micro-organisms are indeed present and to identify them.

## 2 Materials and Methods

### 2.1 X-ray Diffraction (XRD)

The mineralogical composition of all samples was determined with X-ray diffraction (XRD), at the School of Mineral Resources Engineering, Technical University of Crete (Chania, Greece), on a Bruker D8 Advance Diffractometer equipped with a Lynx Eye strip silicon detector, 0.6° divergence and receiving slits, using Ni-filtered CuKaα radiation (35 kV, 35 mA). Data were collected in the 20 range 3–70° 2*θ* with a step size of 0.02° and counting time 1 s per strip step (total time 63.6 s per step). The XRD traces were analyzed and interpreted with the Diffrac Plus 13 software package from Bruker, Germany, and the Powder Diffraction Files (PDF). The quantitative analysis was performed on random powder samples (side loading mounting) by the Rietveld method using the BMGN code (Autoquan^©^ software package version 2.8, Seifert GmbH & Co, Ahrensburg, Germany). About 1 g of finely ground sample <10 μm in size was used for the analyses.

### 2.2 DNA Sequencing

Quantitative polymerase chain reaction (qPCR) was conducted using primers to target the V4 region of the bacterial 16S-rRNA gene (ubiquitous gene used in phylo-taxonomy [26,27]; chloroplast [28]; 18S-rRNA gene of fungus [16,29] and Eurotiales order (e.g., *Talaromyces* spp. and *Penicillium* spp., not well recognized by previous fungal primers; [16]). Each 25-μL PCR reaction mixture consisted of 2.5 μL of diluted DNA sample, 12.5 μL Go-Taq^®^ qPCR Master Mix kit (Promega, Madison, WI; consisting of 1.5 mM MgCl_2_, Taq DNA polymerase, 2x proprietary PCR buffer, 200 mM of each dNTP, and CRX dye), 2.5 μL 10x-primer mixture (0.2 μM final concentration of each primer). Reaction conditions were as follows on a BioRad iCycler5 (BioRad, Hercules, CA, USA) instrument: 3-min initial denaturation (94 °C); 30 cycles of: denaturation (30 s at 94 °C), primer annealing (30 s at temperatures specific for each assay: 58 °C for 16S-rRNA and chloroplast, and 60 °C for *Talaromyces*), and product extension (1 min at 72 °C); and a final extension (10 min at 72 °C). When completed, the instrument maintained the samples at 8 °C. The mixture has a proprietary dsDNA-binding dye to monitor reactions quantitatively [30].

DNA was extracted from 0.25 g of sample material (#01405 and #01406) using Qiagen PowerSoil Extraction Kit (Hilden, Gremany) according to manufacturer’s instructions, and screened quantitatively via UV micro-spectrophotometry (i.e., DNA absorption at 260 nm versus 280 nm for the background). Further details of DNA extraction and handling of samples can be found in [26].

### 2.3 Bacterial Strains

The bacterial strains used in this study were representative Gram-positive and Gram-negative indicators, which are usually employed for the assessment of antimicrobial properties of various materials. Specifically, *Staphylococcus aureus* NCTC 12493 (Gram-positive) and *Pseudomonas aeruginosa* NCTC 10662 (Gram-negative) were chosen for the evaluation of the samples’ bioactivity. Both bacteria were cultured on LB agar (Lab M-Neogen Culture Media) and LB broth (Lab M-Neogen Culture Media) and the desired bacterial concentration in each experiment was adjusted photometrically based on the McFarland scale. Those bacterial species were selected because of their relation to public health issues, as carriers of diseases, and their use as valuable bacterial indicators.

### 2.4 Antimicrobial Tests

Aqueous leachates of the samples were prepared, so as to assess any generated bioactivity. Samples were mixed with deionized water and ultrasonication was performed for 30 min at 25 °C (Julabo ultrasonic bath) followed by centrifugation at 10,000 *g* for 15 min for solids’ removal. The leachate was sterilized in the autoclave (20 min, 120 °C) and was stored for further antimicrobial testing.

Bioactivity of all samples was studied using the broth microdilution method and estimating the Minimum Inhibitory Concentration (MIC). MICs were measured labelling 96-well sterile microtiter trays with dilutions of each sample. The tested concentrations of the samples were in the range of 200–1.6 mg/mL and the bacterial density in each case was 10^5^ CFU/mL. Microtiter trays were incubated at 37 °C for 18–24 h, followed by optical density measurement at 630 nm, using a microplate reader (Labtech LT-4000 Plate Reader) and Manta LML software provided by LabTech International for LabTech LT-4000 Plate Reader, Uckfield, UK.

## 3 Results

### 3.1 XRD

With the exception of sample 01277, the earths have comparable mineralogical composition consisting mainly of clay minerals, mostly kaolinite and quartz, minor illite and trace K-feldspar, plagioclase and anatase (Table 1). In addition, samples 01405 and 01629-1 contain minor goethite and hematite which explain their red/brown color (Figure 1a,f). The presence of hematite and goethite explains the slightly elevated background of 01405 and 01629-1 compared to the remaining samples. The background was modeled adequately during analysis and did not affect quantitative analysis (see Figure S1). 01405 contains also trace chlorite. Sample 01627 has slightly different clay mineralogy as it contains also abundant illite in roughly equal proportions as kaolinite as well as trace siderite and sulfate minerals, namely alunite and anhydrite. Sample 01277, the powder of Terra Tripolitania, has a distinct mineralogy as it consists of dolomite, calcite, and quartz with minor kaolinite and illite and trace K-feldspar plagioclase and jarosite.

### 3.2 Antibacterial Activity

The bioactivity of the samples was tested using their leachates and estimating their MIC against two representative bacterial indicators, namely, *P. aeruginosa* and *S. aureus.* Based on the respective tests performed for the activity of antibiotics, we assessed the inhibitory concentration of the samples that was sufficient for the reduction of 60% of the concentration of the population of each strain (MIC_60_). The derived results are shown in Table 2, according to which the majority of the samples did not exhibit any substantial antimicrobial activity. Samples 01406, 01629-1 and 01629-2 achieved low levels of bacterial reduction and only their high concentration (200 mg/mL) led to bacterial inactivation up to approximately 40% of the bacterial initial load. This result is referred mainly to *S. aureus*, which proved to be slightly more sensitive under the current experimental conditions, despite the fact that it is a Gram-positive bacterium, and therefore more resistant to environmental stressed conditions. This negligible superiority of the Gram-negative *P. aeruginosa* could be attributed to a certain interaction, possibly a chemical one, between the samples’ components and the Gram-positive bacterial strain (*S. aureus*). For following the bacterial reduction (in %) as a function of concentration for each sample see Figure S2).

Sample 01627 resulted in negligible bacterial reduction, while the only sample with considerable activity was 01405, since (a) it was active even at low concentrations (e.g., 12.5 mg/mL) and (b) it had similar behavior towards both bacterial species. The level of bacterial inactivation was increased when higher concentrations of the sample were used, and complete bacterial decay was achieved when the sample concentration was 200 mg/mL. Sample 01277 was mildly antibacterial against *S. aureus* only (100 mg/mL).

### 3.3 DNA Detection

The amount of DNA material extracted from sample materials was below detection (0.6 ng/g-material), with absorbances at 260 nm and 280 nm < 0.001. Although values fell below the range for quantification, PCR amplifications were still attempted. Unfortunately, none of the assays for bacterial, fungal, or chloroplast DNA revealed any detectable signals greater than analytical backgrounds. As such, none was detected in the samples. This may suggest that the samples had no exposure to any biological agents, but it remains a possibility that DNA and/or naturally organic matter have degraded.

## 4 Discussion

Medicinal earths are a little researched but important archaeological resource. They formed an integral part of ancient and more recent pharmacopoeias. MacGregor [8] outlined the history of use of these earths (bole or Terra Sigillata) as a trajectory from ’wonder drug to folk remedy’ and as such they deserve to be investigated further with modern analytical techniques. So far, their study has been approached from a number of different perspectives: (a) historical, as natural materials used to cure various ailments; (b) archaeological, as catalogued artefacts in museum collections, (c) chemical and mineralogical, as clay minerals (kaolinite/montmorillonite/illite) of which they largely comprise, and recently (d) for their selective bioactivity against specific pathogens (as antibacterials or antifungals). There is considerable research on the antibacterial properties of kaolins and smectites [31,32]. However, most of this work has been undertaken on samples from modern clay deposits and not on small quantities removed from historical samples with an acclaimed use over centuries. Can the study of medicinal earths contribute to antibacterial clay research?

In this paper we argue that medicinal earths can potentially bridge gaps in our information about the nature and function of antibacterial clays. This is because as archaeological artefacts they have a long history of use and associated efficacy. Our work over the last few years has been dedicated to the investigation of the antibacterial properties of some medicinal earths of the Aegean, like Samian Earth [33] and Lemnian Earth [15,16]; also clay-iron oxides composites (*miltos*) [26], as well as alunogen and alunite with kaolinite minerals [34]. For example, we demonstrated that antibacterial properties of Samian Earth may have been attributed to its smectite being naturally enriched in Boron [33].

In this contribution, we have analyzed six earths mineralogically and tested them for bioactivity against two pathogens, *P. aeruginosa* and *S. aureus.* Of the six, only one was found to be bioactive against both. This was the red earth 01405, which is presumed be an Armenian bole. We have compared this bioactive sample with bioactive earths from Lemnos (Figure 1i,j) [15,16]. Lemnian Earth 700.17 (red) contained 37.6% kaolinite, 41% illite, 17.7% quartz, and 3.8% hematite. Lemnian Earth 700.18 (yellow grey) contained 66% montmorillonite, 18.1% illite, 6.9% quartz, 9% albite. Of the three, it is 700.17 that is most the similar to 01405, being kaolinite-rich [15]. It is noteworthy that the medicinal earths examined in this study are essentially smectite free.

Comparing the bioactivity of sample 01405 with those obtained from samples 700.17 and 700.18 [16], it seems that sample 01405 exhibited higher antimicrobial activity (MIC_60_ = 12.5 mg/mL) for both bacteria compared to 700.17 and 700.18. Regarding S. *aureus*, MIC_60_ of 700.17 and 700.18 were 10 mg/mL and 20 mg/mL, respectively [16]. By contrast, and regarding *P. aeruginosa*, a higher concentration was required of the Lemnian Earths (MIC_60_ of 700.17 and 700.18 was 50 mg/mL for both). Based on our results, in all cases, *P. aeruginosa* was more resistant in the presence of the tested samples, of which 01405 resulted in higher reduction of bacterial load.

A third sample of Lemnian Earth 700.4 (white) (Figure 1h) contained 65.2% dolomite, 9.9% illite, 17.3% kaolinite, and 7.6% quartz [15]. Sample 700.4 is similar to 01277 despite the latter being more yellow than white; this is presumably on account of small quantities of jarosite and possibly of trace amounts of goethite, the abundance of which is below the detection limit of the method. Samples 01277 and 700.4 are both rich in calcite/dolomite and with only small amounts of kaolinite.

Both 01277 and 700.4 display some antibacterial activity against *S. aureus* (MIC_60_ of 01277 = 100 mg/mL and MIC of 700.4 = 45 mg/mL) and even lower against *P. aeruginosa* (MIC_60_ of 01277 ≥ 200 mg/mL and MIC_60_ of 700.4 = 90 mg/mL). It is not clear why dolomite might have weak antibacterial properties. It has been reported that dolomite can develop antiviral (rather than antibacterial) properties only after it has been heated between 800 °C and 1400 °C [35]. Dolomite in association with gypsum and calcite has been reported in the vicinity of Garyan, in the region of Tripolitania, N.W. Libya [36] (p. 35), suggesting a Libyan source for sample 01277.

In a forthcoming publication we have argued that for the two samples of Lemnian Earths (700.17 and 700.18), the driving force behind their bioactivity may be attributed to their organic load, the secondary metabolites of the fungus *Talaromyces* spp., of the order Eurotiales, which includes *Penicillium* spp. [16]. We also concluded that bulk clay mineralogy (kaolinite/illite/montmorillonite), on its own, does not appear to drive bioactivity. This is expected in as much as the z-potential of the clay minerals present (kaolinite, illite, chlorite along with smectite), as well as the bacteria tested (S. *aureus* and *P. aeruginosa*) are negative in the pH range 4–10 [37–39]. Therefore, a mutual repulsion between the clay particles and the bacteria cells is expected in the aqueous leachates. However, nanoparticles, other than those of clay minerals, may also have a role to play.

In the same publication [16] and regarding the organic load of samples 700.17 and 700.18, we have suggested that it may have been introduced ‘intentionally’ as deduced from an account of the extraction of the Lemnian Earth provided by Galen [20] (pp. 169–170), albeit quite a few centuries earlier. In the case of the red (Armenian?) bole (01405), examined here, no such organic load was identified, so an alternative ‘driver’ needs to be sought. It is possible that the chemistry of the leachate of 01405 might provide some insight. In this paper we have not been able to provide chemical analysis (major and trace elemental composition) of the leachates of the earths, and in the manner carried out in other studies [16]. Nevertheless, in work carried out so far, the abundances of metalloids, non-metals and heavy metals in the leachates (e.g., B, Al, As, Hg, Sb, Se, Pb, Cu, Zn, etc.), which would be detrimental to bacterial cells, and thus be in themselves the key drivers of bioactivity, are in the level of a few ppb. Only when the clay is doped or is naturally enriched in one or more of these elements could they be the drivers of bioactivity [15,16,33].

Regarding the Armenian bole, Galen mentions that it originated in a mountain in the city of Bagouana which is a Hellenised rendering of the ancient Armenian Bagawan, a place that was most probably near today’s Diyad in the Turkish Province of Aǧri, very close to the modern Turkish-Armenian border ([40] (pp. 283.11–14); [41] (pp. 160.14–17); [42,43]). Today, one of the local tourist attractions in the area are the Meya caves with an unspecified length of occupation. If there is a starting geographical/geological point in the investigation of Armenian bole, it could be from that locality.

Many earths, in addition to Armenian bole, were thought to be ‘against the plague’, but it is not clear what precisely was meant by the ‘plague’. Regarding the beneficial effects against quartan fever, most probably a type of malaria, as suggested by the 6th century author, Alexander of Tralles, research into the characterization of the secondary metabolites in one sample of Lemnian Earth (700.18) produced some intriguing results. It revealed bioxanthracene, a fungal secondary metabolite [15]. Bioxanthracenes are known to be used as antibiotics and against malaria [44].

As mentioned earlier and based on the analyses presented here, we can provide no explanation for the antibacterial action of sample 01405, although chemical analysis of the leachate might have shed some light. It is not possible to know what effect long term museum storage under environmentally uncontrolled conditions may have had on the antibacterial properties of the original sample. It may have altered existing properties or indeed may have imparted the earth with antibacterial properties when none existed in the first place. Not all medicinal earths circulating in the post-medieval markets of Europe were genuine. Hence, the geochemical/biogeochemical characterization of the museum objects on their own cannot suffice.

As already mentioned in the introduction, medicinal earths are identified primarily by their geographical origin. We suggest research into potentially antibacterial medicinal earths should begin not at the ‘museum’ but in the ‘field’, the original place of extraction of the clays used in the making of these medicinal earths. Not all of these places of origin can be easily identified. Even those localities that are ‘confidently’ known, like Kotsinas on N E Lemnos, the place of the extraction of Lemnian Earth, present the investigator with a whole host of geo-archaeological queries requiring clarifications based on extensive surveying and sampling.

Further to the geology, there is a need to understand human agency. Beneficiation or ‘washing’ as is often referred to in the texts requires an understanding of the relevant practices entailed. Was it simply a case of levigating the extracted clay?

Once select clay deposit localities used in antiquity have been tentatively identified and practices tentatively characterized, then starts the ‘long haul’ of experimental work on both natural clays and archaeological samples, alike. As mentioned earlier, some earths, like Armenian bole, were thought effective in the treatment of infectious diseases causing epidemics. The parallel investigation of (a) earths in museum collections with ‘secure’ provenance and (b) clays from their geographical sources, with a battery of analytical techniques followed with microbiological testing, is highly likely to provide an insight into their antibacterial and potential other properties. These are ambitious and long-term projects and they require the combined efforts of many scholars and from many fields. However, given the ongoing fight against antimicrobial resistance and the need to find potentially ‘new’ agents that prevent bacteria and other microorganisms from developing this resistance, turning to the medicinal earths of antiquity and more recent times, for both insight and solutions, may prove to be a worthwhile task.

## Supplementary Material

**Supplementary Materials:** The following are available online at http://www.mdpi.com/2075-163X/10/4/348/s1, Figure S1: XRD traces of samples 01277 and 01405. Sample 01405 has elevated background due to the presence of hematite and goethite. Figure S2: Reduction of bacterial population of *S. aureus* (Gram-positive) and *P. aeruginosa* (Gram-negative) after incubation with the tested samples.

Supplementary Materials

## Figures and Tables

**Table 1:** X-ray diffraction (XRD) results of minerals present within each of the six earths. Composition in weight %.

Minerals	01277	01405	01406	01627	01629-1	01629-2
Calcite	34.5					
Dolomite	22.1					
Gypsum	1.5					
Illite	6.9	4.5	7.2	33	13.1	3.3
Jarosite	0.6					
Kaolinite	5.2	49.7	54.1	26	45.4	66.5
K-feldspar	2.8	2	2.6	3.8		1.9
Plagioclase (albite)	2.4	1.2	2.4	1.2	1.1	1.6
Quartz	24	27.2	30	32	28.1	26.1
Goethite		8			3.7	
Hematite		5.1			7	
Chlorite		0.7	2.7			
Anatase		1.6	1	0.4	1.1	0.6
Siderite				0.6	0.5	
Alunite				1.3		
Anhydrite				1.7		

**Table 2:** MIC_60_ values of the tested samples against *S. aureus* and *P. aeruginosa*.

Sample	MIC_60_ (mg/mL)	
	*S. aureus*	*P. aeruginosa*
01277	100	>200
01405	12.5	12.5
01406	>200	>200
01627	>200	>200
01629-1	200	>200
01629-2	>200	>200
