# Supplementary material for: Bridging the Gaps: Bole and Terra Sigillata as Artefacts, as Simples and as Antibacterial Clays
Source: Minerals (Basel). Author manuscript; Available in PMC 2020 Jul 28. (PMC7115821; doi:10.3390/min10040348)
Supplement: Supplementary Materials [file EMS86262-supplement-minerals_10_00348_s001.pdf]

Supplementary Materials

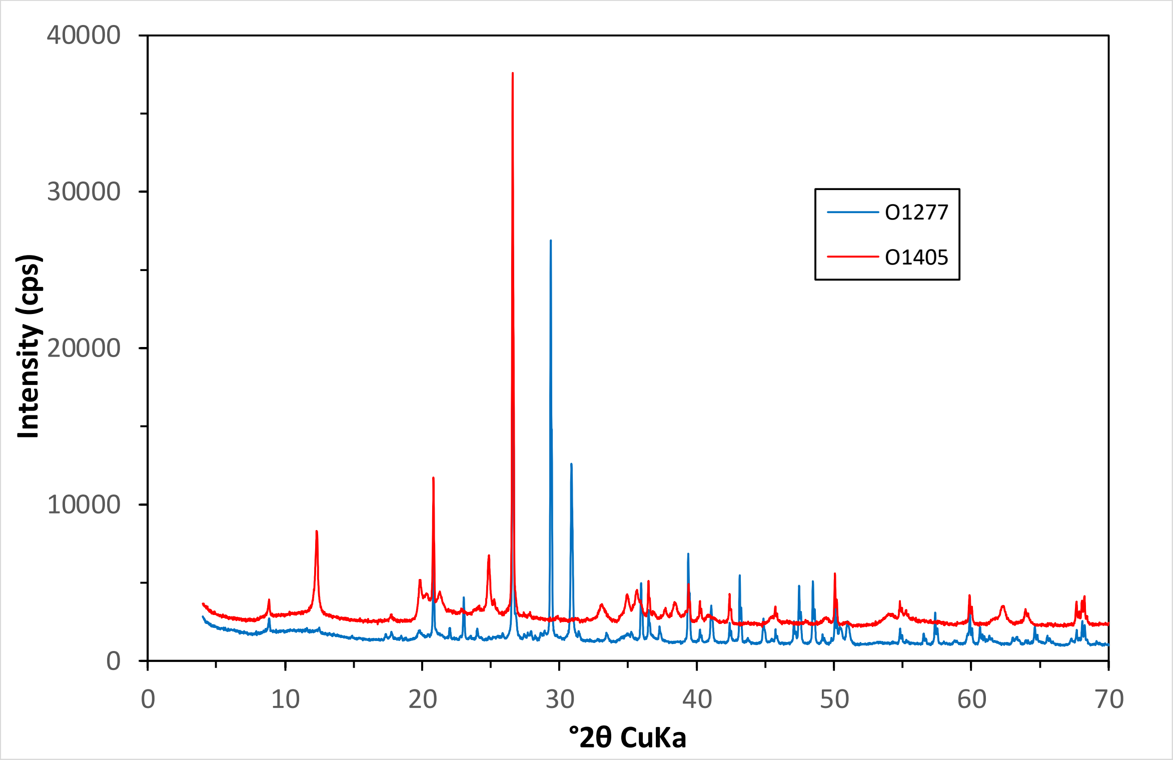

Figure S1. XRD traces of samples 01277 and 01405. Sample 01405 has elevated background due to the presence of hematite and goethite.

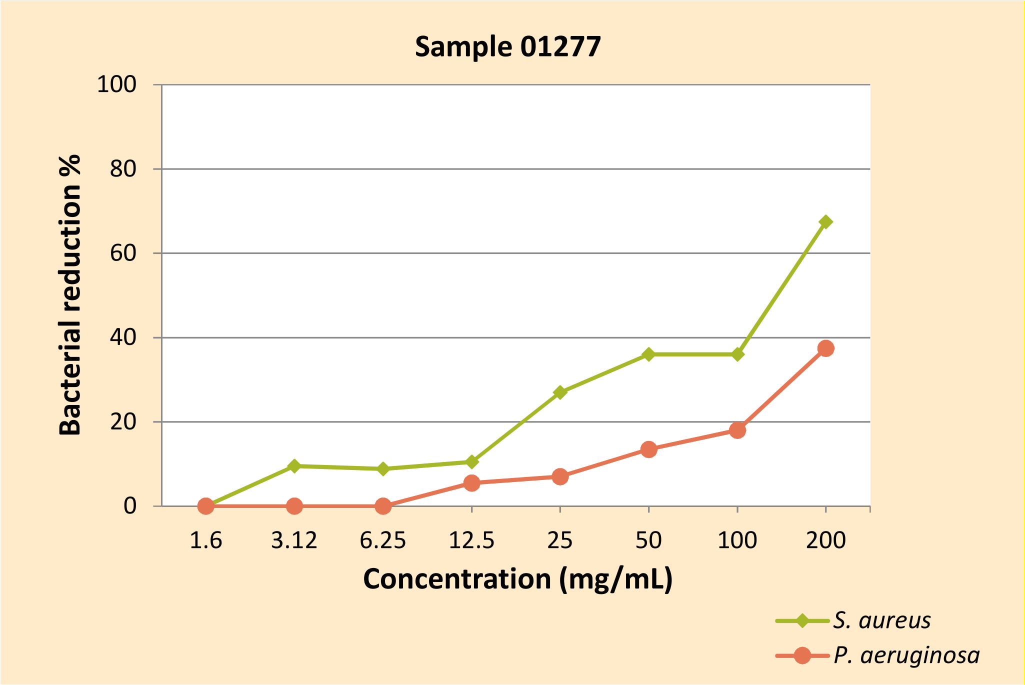

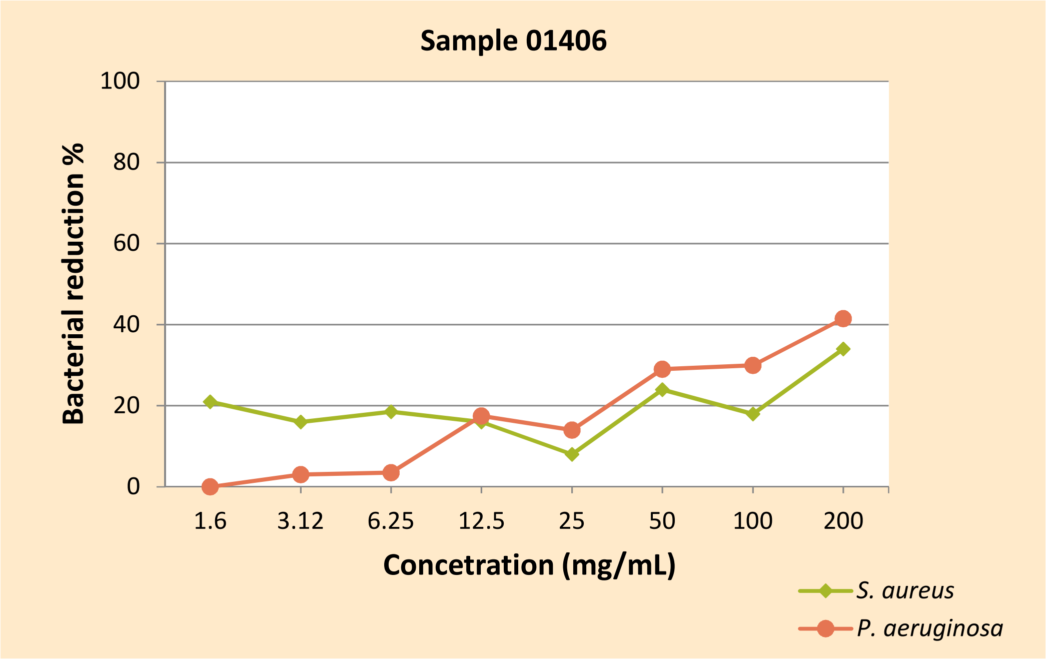
Sample 01406
Bacterial reduction %
100
80
60
40
20
0
1.6
3.12
6.25
12.5
25
50
100
200
Concetration (mg/mL)
S. aureus
P. aeruginosa

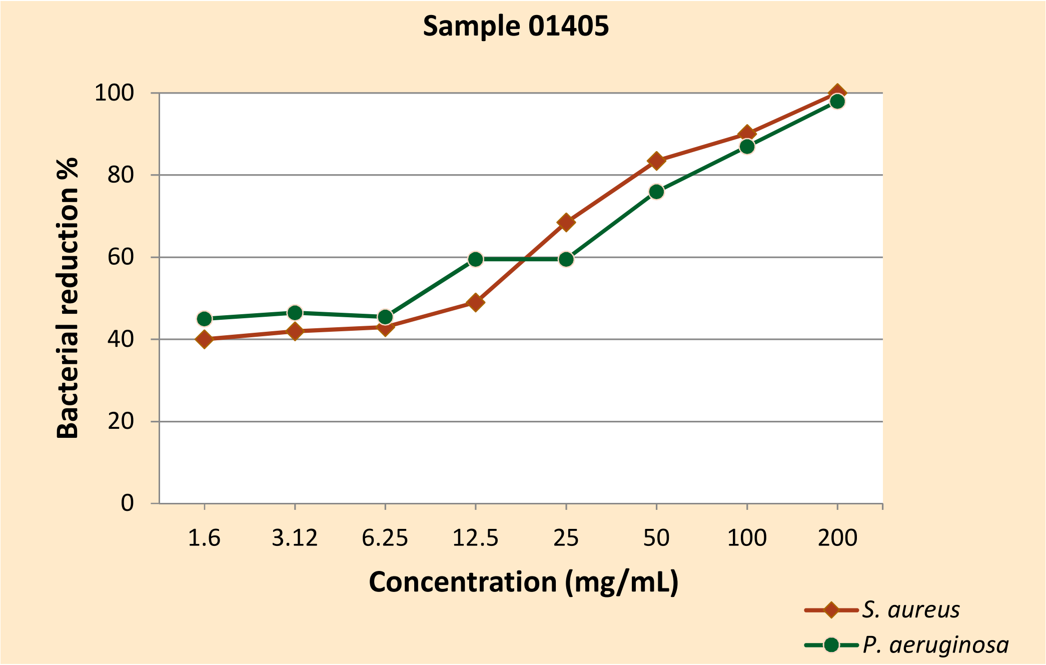
Sample 01405
Bacterial reduction %
100
80
60
40
20
0
1.6
3.12
6.25
12.5
25
50
100
200
Concentration (mg/mL)
S. aureus
P. aeruginosa

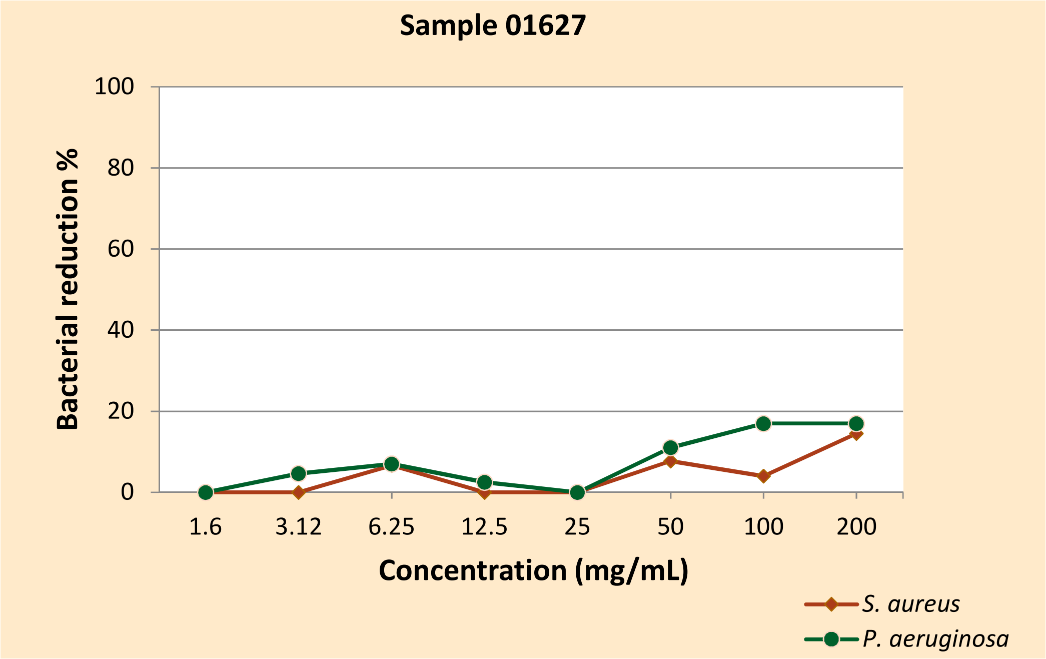
Sample 01627
Bacterial reduction %
100
80
60
40
20
0
1.6
3.12
6.25
12.5
25
50
100
200
Concentration (mg/mL)
S. aureus
P. aeruginosa

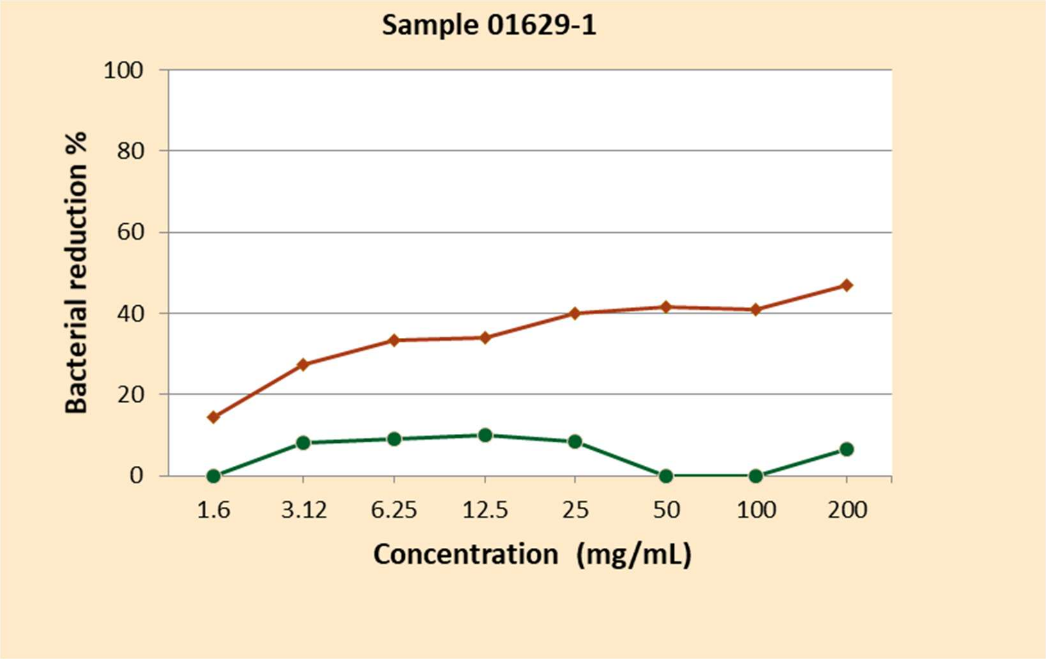
Sample 01629-1
Bacterial reduction %
100
80
60
40
20
0
1.6
3.12
6.25
12.5
25
50
100
200
Concentration (mg/mL)

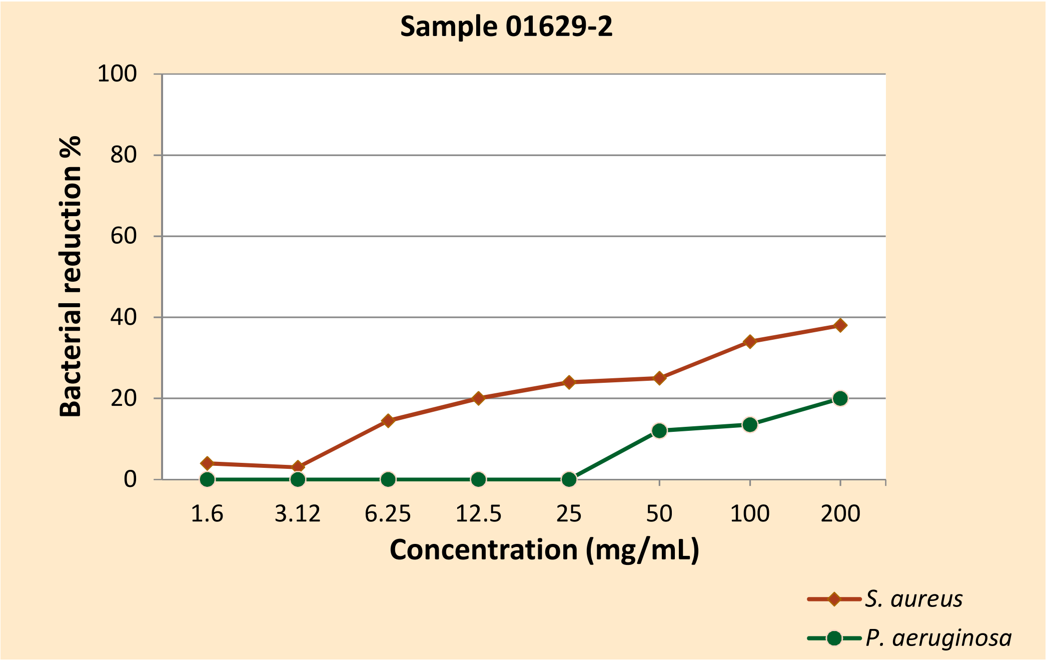

Figure S2. Reduction in % bacterial counts as a function of concentration, on a sample by sample basis and for G-Positive *S. aureus* and Gram-negative *P. Aerugenosa.*
